# The Sensory Gatekeeper of the Larynx: Anatomy and Clinical Importance of the Internal Branch of the Superior Laryngeal Nerve

**DOI:** 10.3390/diagnostics15131711

**Published:** 2025-07-04

**Authors:** Alexandra Diana Vrapciu, Iulian Brezean, Răzvan Costin Tudose, Mugurel Constantin Rusu, George Triantafyllou, Maria Piagkou

**Affiliations:** 1 Division of Anatomy, Department 1, Faculty of Dentistry, “Carol Davila” University of Medicine and Pharmacy, 020021 Bucharest, Romania; alexandra.vrapciu@umfcd.ro (A.D.V.); razvan-costin.tudose0721@stud.umfcd.ro (R.C.T.); 2 Department of General Surgery, “Carol Davila” University of Medicine and Pharmacy, Cantacuzino Clinical Hospital, 030167 Bucharest, Romania; iulian.brezean@umfcd.ro; 3 Department of Anatomy, Faculty of Health Sciences, School of Medicine, National and Kapodistrian University of Athens, 11527 Athens, Greece; georgerose406@gmail.com (G.T.); mapian@med.uoa.gr (M.P.)

**Keywords:** internal branch of the superior laryngeal nerve, larynx, superior laryngeal nerve, superior laryngeal artery, vagus nerve

## Abstract

The internal branch of the superior laryngeal nerve (IbSLN) plays a critical role in the sensory innervation of the supraglottic larynx. It is essential for protective reflexes such as coughing and swallowing. This nerve is frequently at risk during surgeries involving the cervical region, including thyroidectomy, carotid endarterectomy, and anterior cervical spine procedures. Injury to the IbSLN may lead to postoperative complications. A comprehensive review of the morphological and topographic characteristics of the IbSLN is presented, focusing on its anatomical course, relationships with key vascular structures, branching patterns, and clinically significant variations.

## 1. Introduction

The vagus nerve gives rise to several important branches in the neck [1,2], among which the superior laryngeal nerve (SLN) is particularly interesting due to its role in voice modulation and airway protection [3]. After exiting the vagus nerve inside the carotid sheath, the SLN descends and divides into two branches of different calibre: the internal branch of the superior laryngeal nerve (IbSLN), the larger branch, and the external branch of the superior laryngeal nerve (EbSLN), the smaller one [4,5]. Apart from the calibre, the two branches are also differentiated by direction; the IbSLN runs more transversely than the EbSLN [6]. In rare instances, both the IbSLN and EbSLN leave directly from the vagus nerve immediately below the inferior vagal (nodose, plexiform) ganglion or at the ganglion level [7,8]. This variant, in which there is no common trunk of the SLN and the two branches arise directly from the inferior ganglion of the vagus nerve, has been reported in 5% [9] or 6% [8] of cases.

**Figure 1:**
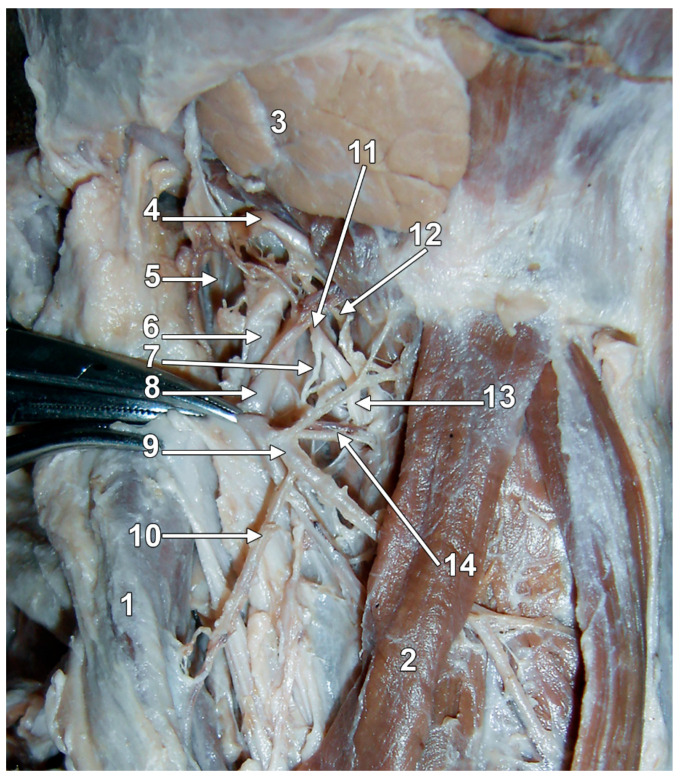
Original dissection of the right carotid triangle, infero-lateral view. 1. Sternocleidomastoid muscle; 2. sternohyoid muscle; 3. submandibular gland; 4. hypoglossal nerve; 5. internal jugular vein; 6. internal carotid artery; 7. external branch of the superior laryngeal nerve; 8. external carotid artery (reflected); 9. superior thyroid artery; 10. sternocleidomastoid branch; 11. superior laryngeal nerve; 12. nerve of the thyrohyoid muscle; 13. internal branch of the superior laryngeal nerve; 14. superior laryngeal vessels.

Anatomically, the IbSLN follows a consistent yet intricate course from its origin to its entry into the larynx through the thyrohyoid membrane (THm), often accompanied by the superior laryngeal artery (SLA) [10,11,12] (Figure 1). Sympathetic fibres from the superior cervical ganglion join the SLN and/or any of its branches; likely, they play a role in laryngeal function, possibly influencing mucus secretion and blood vessel regulation within the larynx [8].

Given its slender calibre and variable branching pattern, the IbSLN is particularly susceptible to injury during neck surgeries [11]. Damage to this nerve can lead to impaired laryngeal sensation, absent cough reflex, silent aspiration, and phonatory dysfunction [13,14].

This review aims to consolidate the anatomical knowledge of the IbSLN, emphasising its clinical relevance, common anatomical landmarks, and risk zones during surgical procedures. Enhancing awareness of the IbSLN’s course and variability is essential for preserving the laryngeal function and reducing complications in cervical surgeries.

## 2. Literature Search and Selection Criteria

A comprehensive literature search was conducted using PubMed, Scopus, and Google Scholar databases. Search terms included combinations such as “internal branch of the superior laryngeal nerve”, “superior laryngeal nerve”, “laryngeal sensory innervation”, “nerve injury larynx”, and “laryngeal nerve anatomy”. The search included English-language publications with no exclusion based on publication date, allowing the incorporation of foundational anatomical studies dating back to the 1920s through June 2025. Eligible sources encompassed cadaveric studies, imaging-based anatomical analyses, clinical investigations, and surgical case series. Exclusion criteria included non-English articles, studies unavailable in full-text PDF format, low-quality publications characterised by inadequate methodological descriptions or irrelevant findings, studies published in unindexed or low-impact journals, and research conducted on fetal specimens. Additional relevant sources were identified through manual screening of reference lists from key publications. Although a formal quality assessment was not performed, all included studies were evaluated for relevance, anatomical detail, and their overall contribution to understanding the structure, function, and clinical significance of the IbSLN.

## 3. The Gross Anatomy of the IbSLN

After emerging from the SLN at the level of the hyoid bone [15,16,17], the IbSLN descends and passes just inferior to the greater horn of the hyoid bone (GhHb) [4]. At this point, the IbSLN changes direction and runs medially [11]. The antero-infero-medial course of the IbSLN nerve was demonstrated by Kiray, who measured two distances for this purpose: the distance between the IbSLN and the midline, and the distance from the anterior surface of the intervertebral disks and the IbSLN, at different vertebral levels, in a dissection study on 12 cadavers [11]. Thus, at the C2–C3 intervertebral disk level, the distance from the midline is 24.2 ± 3.3 mm, at the next intervertebral disk level, the distance is 20.2 ± 3.6 mm, and at the C4–C5 level, the distance is the smallest, i.e., 15.9 ± 4.3 mm [11]. As the IbSLN descends, it moves away from the cervical spine, and its distance from the intervertebral discs increases, specifically from 9.6 ± 3.2 mm at the C2–C3 disc to 23.0 ± 5.2 mm at the C4–C5 intervertebral disc [11]. The anterior, inferior, and medial direction of the IbSLN was confirmed by a dissection study in 36 human cadavers [18].

The course of the IbSLN close to the GhHb provides a consistent anatomic landmark for clinicians and anatomists [11,19,20]. Quantitative measurements have been documented in anatomic studies, indicating that the distance from the point where the IbSLN crosses the GhHb to the point of thyrohyoid membrane (THm) piercing averages approximately 25.8 ± 5.5 mm [11], 28.52 ± 4.61 mm [21], or 20.00 mm ± 5.0 mm [22]. The distance from the GhHb to the termination point of the IbSLN middle branch was also recorded as 25.8 ± 5.5 mm [11]. The IbSLN descends, making an angle of about 49° with the tracheoesophageal sulcus [23]. Along its course, the IbSLN is always medial to the facial and lingual arteries [18,23]. Toward the THm, it travels alongside the SLA, a branch of the superior thyroid artery (STA). Before piercing the THm to enter the larynx, the IbSLN passes deep to the thyrohyoid muscle [10]. Regarding the superior cervical ganglion, the IbSLN is ventral to it in 66.67%, and medial to it in 33.33% [11].

## 4. The Origin Level of the IbSLN

Regarding the emergence of the IbSLN from the SLN, its origin was located either deep to the internal carotid artery, about 35 mm from the carotid bifurcation [11,24], or deep to the carotid bifurcation [25]. The cervical level of the origin of the IbSLN has been investigated in several cadaveric studies [11,26]. The IbSLN most commonly leaves the SLN at C2 [11,26] (Table 1).

## 5. The Relations Between the SLA and the IbSLN

The IbSLN courses are immediately inferior to the GhHb and approach the THm, typically accompanied by the SLA, a branch of the STA [27,28]. This close anatomical relationship between the IbSLN and the SLA holds significant clinical relevance during cervical procedures, drawing considerable interest from anatomists and surgeons [23,29]. In a cadaveric study involving 14 specimens, it was found that in over 64% of cases, the IbSLN was positioned supero-posteriorly to the SLA. In contrast, in the remaining cases, it was located infero-posteriorly to the artery [29]. Another study on 50 cadavers reported that the IbSLN was inferior to the SLA in only 11% of cases [23]. Similarly, a different anatomical exploration revealed that in nearly 60% of specimens, the IbSLN was superior to the SLA, and at the point of penetration through the THm, the nerve was situated medially to the artery [30]. A report based on the dissection of 24 sides demonstrated that in 25% of cases, the IbSLN did not cross the SLA, while in the remaining 75%, it crossed the SLA approximately 31.4 mm superior to the upper pole of the thyroid gland [11]. Regarding its relation to the STA, the IbSLN did not cross this artery in 62.5% of cases; in the 37.5% of cases where it did, the crossing occurred at an average distance of 33.0 mm above the upper pole of the thyroid gland [11]. A dissection study indicated that the IbSLN is parallel to the SLA; in 88.89% of cases, the IbSLN was placed superior and posterior to the SLA, while in 11.11%, it was inferior and posterior [18]. Another cadaveric study, focusing on the variable anatomy of the STA, indicated that the SLA descends to the larynx with the IbSLN nerve superior to it [28]. This study, conducted through dissections in 20 cadavers, found that the origin of the STA may be higher, lower, or at the same level as the GhHb [28]. The STA may course laterally, medially, or posterior to the GhHb [31]. As the STA’s topography may vary, the IbSLN-to-SLA placement should also be variable. Therefore, surgeons should carefully dissect them, and a pre-determined nerve-artery topography should not be assumed. A dissection study on 14 sides of 7 fresh-frozen and silicone-injected cadavers revealed that the two anatomical structures, the IbSLN and the SLA, are closest to each other near the superior horn of the thyroid cartilage [10]. However, the authors did not indicate the exact location of the IbSLN along the SLA [10]. The distance between the two was 5.78 ± 0.36 mm on the right and 5.98 ± 0.48 mm on the left [10], showing proximity and a potential risk of nerve injury during surgical procedures.

## 6. The Location of the Penetration Point of the IbSLN

Although it is most commonly described that IbSLN crosses the THm along with the SLA through a shared orifice placed in the postero-inferior portion of the membrane [32,33,34,35], a dissection study found that IbSLN invariably penetrated the THm within 10 mm (superior) of SLA [6], a result supported by another source [36]. Only one study assessed the position of the penetration point relative to the THm midline; however, the results are not particularly enlightening [26]. The study found that the IbSLN pierces the THm: medial to the midline of the THm in 50% of specimens, lateral to the midline of the THm in 46%, and in the remaining cases, the nerve could not be traced [26].

Because precise knowledge of where the internal branch of the IbSLN penetrates the THm is crucial for surgical safety and success in neck procedures, several studies have placed the entry of the IbSLN nerve through the THm in relation to various cervical anatomical landmarks (Table 2). Accurate localisation of the penetration point enables surgeons to establish safe dissection planes and avoid zones of high nerve injury risk. By understanding the relationship between the penetration point and anatomical references, such as the hyoid bone, thyroid cartilage, and vascular structures, surgeons can preserve laryngeal function.

In addition, real-time identification of the penetration point during surgery provides crucial spatial orientation, enabling informed decisions about tissue handling, the use of electrocautery, and the extent of dissection.

Regarding the penetration of the IbSLN through the THm in relation to the cervical spine, it has been reported that it is frequently found at the C4 vertebra or in neighbouring intervertebral spaces (Table 3). Consequently, the IbSLN is highly vulnerable during high cervical spine surgeries. Given its tendency to pierce the thyrohyoid membrane around the C3–C5 levels, the IbSLN should be carefully considered in preoperative planning and intraoperative dissection.

The clinical imperative for understanding the anatomy of the IbSLN penetration point cannot be overestimated—it can be the cornerstone of successful nerve preservation.

## 7. The Relationship of the IbSLN with the Thyroid Foramen

The thyroid foramen (TF) represents a clinically significant anatomical variant, characterised by a defect in the thyroid cartilage of the larynx, which occurs in over 24% of individuals, with bilateral presentation in approximately 6% [38,39]. Understanding TF anatomy is crucial for surgeons because it consistently contains neural communications that can complicate intraoperative nerve identification and increase the risk of inadvertent injury.

The neural component within the TF is predominantly an anastomotic branch between the IbSLN and EbSLN, consistently reported across multiple studies, with a prevalence ranging from 75% to 100% of cases [36,40,41,42,43]. This anastomosis may occur alone or in conjunction with vascular elements [40,44,45], forming a complex neurovascular bundle. Recognition of this anatomical variant is therefore essential for successful nerve preservation strategies during cervical procedures.

## 8. The Branching Pattern of the IbSLN

**Figure 2:**
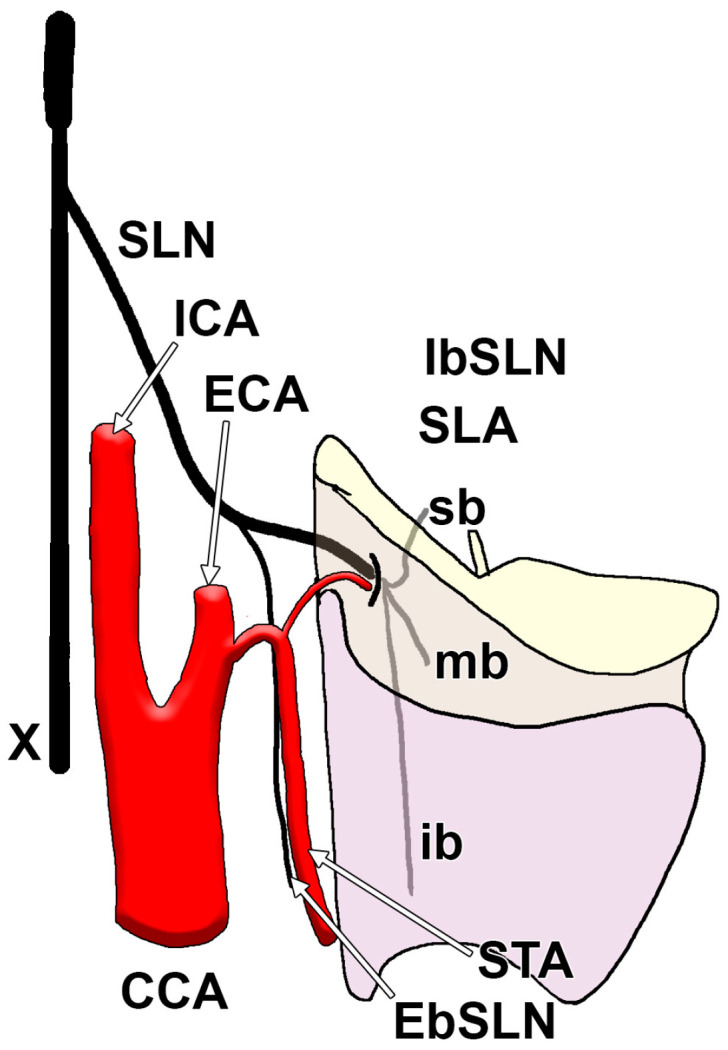
Drawing of the right superior laryngeal nerve (SLN). Lateral view. X: vagus nerve; CCA: common carotid artery; ICA: internal carotid artery; ECA: external carotid artery; STA: superior thyroid artery; SLA: superior laryngeal artery; sb: superior branch; mb: middle branch: ib: inferior branch; IbSLN: internal branch of the SLN; EbSLN: external branch of the SLN.

The IbSLN exhibits notable anatomical variability in its typical branching pattern (Figure 2), which carries significant implications for surgical procedures involving the neck, particularly those involving the thyroid and laryngeal areas. Most anatomical descriptions report a trifurcation of the IbSLN after it pierces the THm, dividing into superior, middle, and inferior branches [11]. However, variability in the number and site of these branches has been documented across multiple dissection studies (Table 4).

While post-THm trifurcation is frequently described [44,45], even in anatomical texts [7], pre-THm branching is also common, with its prevalence potentially reaching 100% [18], which challenges the traditional teaching of post-membrane trifurcation. This variance likely depends on factors such as study design, dissection methods, and ethnicity.

Thus, the IbSLN demonstrates significant anatomical variability that directly impacts surgical safety in thyroid and laryngeal procedures. Surgeons must be prepared for two critical variations: the nerve may branch either before or after piercing the THm, and the number of branches can range from two to five. The presence of extralaryngeal branching, where the nerve divides before entering the larynx, underscores the importance of meticulous surgical dissection and awareness of anatomical variations to prevent nerve injury and preserve laryngeal function. Early branching increases the risk of inadvertent injury during dissection, as multiple nerve branches must be identified and protected rather than a single trunk.

These findings mandate meticulous surgical technique with systematic identification of all nerve branches, regardless of their relationship to the THm, to prevent adverse events.

## 9. The Branches of the IbSLN

Based on their course and distribution, the primary branches of the IbSLN were classified as superior, middle, and inferior [21]. Up to a maximum of 13 secondary branches have been recorded [44].

The upper branch supplies the epiglottis and the lateral glossoepiglottic fold, making a nervous plexus on the posterior side of the epiglottis [35,44,45].

The middle branch, 22.68 ± 4.07 mm long, supplies the mucosa of the aryepiglottic fold, the vestibule, the true and false vocal folds, the ventricle, and the mucosa covering the arytenoid cartilage [21,35]. Adjacent to the true and false vocal folds, the branches of the middle division form a consistent nerve plexus [45]. It is assumed that the middle branch of the IbSLN constitutes the afferent pathway of the cough reflex [21].

The inferior branch of the IbSLN, the largest and thickest of the three, supplies the aryepiglottic fold, the ventricular mucosa, and the mucosa of the arytenoid cartilage, extending to the infraglottic mucosa and the hypopharynx [21,35,45]. Usually, branches of the inferior division join with the recurrent laryngeal nerve’s (RLN) posterior branches to form Galen’s anastomosis [44,45].

Considered by many to be a sensory nerve [15,46,47,48,49], the IbSLN is, in fact, a mixed nerve. It provides mucosal sensory innervation to the supraglottic larynx’s mucosa, including the epiglottis and laryngeal inlet. This sensory input is crucial for triggering protective reflexes, such as the glottic closure and coughing [30]. Some evidence indicates that the IbSLN provides motor innervation of the interarytenoid muscles [50,51,52,53]. Sometimes, the IbSLN supplies the superior oblique fibres of the inferior constrictor muscle of the pharynx [54].

Different opinions have arisen to suggest that the IbSLN may supply part of the motor fibres to the adductor muscles of the larynx, especially the interarytenoid muscle. However, a microdissection study on 12 human larynges found that all branches of the IbSLN entering the interarytenoid muscle perforated it, and none terminated within the muscle [44]. Mu et al. (1994) used ten human adult larynges on which they applied Sihler’s stain and demonstrated that the interarytenoid muscles received branches from the IbSLN [51]. Four years later, Sanders and Mu used Sihler’s stain to study the SLN in five human larynges and supported the previous evidence of the IbSLN supply of the interarytenoid muscle [45].

## 10. The Anastomoses of the IbSLN

Through their branches, the laryngeal nerves can provide various anastomoses [55,56,57,58,59]. One of the most constant and commonly documented connections is Galen’s anastomosis, or ansa, which links the posterior branches of the IbSLN to the RLN [60]. Ónodi (1902), quoted in Rueger (1972), described that a branch of the IbSLN “runs downward on the posterior surface of the cricoarytenoideus posticus and fuses with the RLN, forming the so-called Ansa Galeni (ramus communicans or anastomoticus)” [44,61]. Onodi also found smaller anastomoses between the IbSLN and RLN in or about the interarytenoid muscle [44,61]. They were connected to Galen’s ansa and/or a perforating branch of the IbSLN [44,61]. Three different anastomotic possibilities were listed: (1) across the posterior surface of the interarytenoid muscle, to the motor branch of the RLN for this muscle; (2) an intramuscular anastomosis of the IbSLN and RLN inside the interarytenoid muscle—a deep ansa, further sending off twigs to the muscles and the mucosa; and (3) fibres of the IbSLN reach deep to that muscle on the laryngeal mucosa, converge towards the midline, cross it, and connect with the RLN [44,61].

A dissection study found this nervous communication in 17 out of 19 laryngeal sides and identified Galen’s ansa with a double-loop configuration in 2 sides [44]. The arytenoid plexus involves fibres from the anterior branch of the RLN joining with the arytenoid branch of the IbSLN [62]. A recent meta-analysis indicated a pooled prevalence rate of 79.7% for the arytenoid plexus [63]. Its preservation is considered crucial for the synchronised movement of the vocal folds and effective phonation. Another relevant neural link is the thyroarytenoid communication, formed between a descending branch of the IbSLN and an ascending branch from the RLN [43,64]. Communications between the IbSLN and the EbSLN are commonly seen passing through the TF [39]. Complex or duplicated anastomotic patterns have also been described, such as an atypical double-loop configuration, where one neural loop connects the IbSLN to the EbSLN, and the second is the ansa of Galen that joins the EbSLN to the RLN [65].

The laryngeal nerves’ anastomoses should be considered in case of suspected laryngeal nerve injury; if present, the anastomoses can lead to an unexpected abnormal position of the vocal cords [63].

The clinical importance of the SLN in thyroid surgery has received significantly less attention than that of the RLN [66]. An injury to the EbSLN is a voice-altering complication of thyroid surgery that has significant implications for professional voice users [66]. The symptoms of EbSLN injury can be nonspecific, and the subtle laryngoscopic manifestations are often overlooked [66]. Video-laryngoscopic examination in an EbSLN lesion reveals the absence of contraction of the cricothyroid muscle with normal adduction and abduction of the vocal folds [66]. On the other hand, a normal contraction of the cricothyroid muscle with altered adduction of the vocal fold may raise suspicions of either an IbSLN lesion, an RLN compression, or both. However, the EbSLN and RLN are exposed to risk during thyroidectomies [67], rather than the IbSLN. Anastomoses of the IbSLN and RLN may, however, masquerade as potential lesions of the RLN during thyroidectomy.

## 11. The Identification or Avoidance Areas of the IbSLN

Hill proposed the identification of the IbSLN in a region bounded by the following landmarks: the THm deeply, the carotid bifurcation laterally, the thyrohyoid muscle medially, the GhHb superiorly, and the STA and SLA caudally [25]. Between these landmarks, the IbSLN is found immediately above the superior horn of the thyroid cartilage [25].

The danger zone was also defined. This is the space where the IbSLN is most likely to penetrate the THm [33]. This IbSLN danger zone is the intersection of the following distances: 1.97 ± 0.29 cm lateral to the midpoint of the thyroid notch, 0.60 ± 0.14 cm cranial from the superior border of the thyroid cartilage, 2.04 ± 0.30 cm from the midpoint of the hyoid body, and 1.65 ± 0.38 cm from the GhHb [33]. In this area, surgeons must be meticulous, and dissections must be performed with great care. According to rigorous measurements, 1 cm lateral to the midpoint of the thyroid notch, the dissection is secure [33], and the risk of nerve injury is low.

The potential role of intraoperative ultrasound, autofluorescence, or nerve monitoring may be considered to reduce the risk of injury to the IbSLN. These intraoperative technologies have varying utility for protecting the nerve. Intraoperative ultrasound allows a limited direct visualisation because the IbSLN is small and runs within the thyrohyoid membrane, making direct ultrasound identification challenging. However, it can identify vascular landmarks and tissue planes that guide safer dissection around the superior pole of the thyroid lobe and may help locate the superior thyroid artery. Autofluorescence has theoretical potential because it may highlight nervous tissue; however, the IbSLN’s small calibre and deep location may limit its practical application. Autofluorescence warrants further investigation for the identification of small nerves in thyroid surgery. Intraoperative nerve monitoring is challenging because the IbSLN is regarded as purely sensitive, although some disputes exist regarding this assessment. In this regard, traditional EMG monitoring looks ineffective. Standard intraoperative nerve monitoring relies on detecting muscle contractions, which do not occur with sensory nerve stimulation. Therefore, the best protection currently relies on: (1) anatomical knowledge—understanding the IbSLN’s course; (2) careful dissection technique—gentle handling of tissues around the superior thyroid pole; (3) preservation of fascial planes—maintaining the integrity of the thyrohyoid membrane when possible; and (4) surgical experience. A meticulous surgical technique based on anatomical landmarks remains the primary method for protecting the IbSLN.

## 12. The Injury of the IbSLN

The IbSLN is vulnerable to damage during various surgical procedures, such as carotid endarterectomy [11], hybrid surgery for revascularisation of chronic occlusion of the internal carotid artery (carotid endarterectomy + carotid artery stenting) [68], cervical spine surgery [15,69], thyroidectomy, parathyroidectomy, cervical oncologic procedures [29], transoral robotic surgery, or endoscopic laryngeal procedures [10].

Its risk of injury is due to its relatively great length (Table 5) and small diameter (Table 6).

During surgery, the IbSLN can be injured by inadvertent ligation with vascular structures or accidental cutting during dissection, compression from surgical instruments or retractors, stretching due to manipulation, and thermal injury from cautery devices [6,11,69,72,73].

A plethora of studies discuss, evaluate, and highlight the risk of injury to the external branch of the superior laryngeal nerve [74,75,76,77,78,79,80,81] or recurrent laryngeal nerve [82,83,84] during thyroid surgery. However, the issue of potential iatrogenic injury to the IbSLN during thyroidectomy is rarely brought up [62,72,85]. The IbSLN is prone to injury during thyroid surgery, particularly when dissecting near the superior pole of the gland. The risk of damage to the IbSLN increases with large goiters when the upper pole of the gland ascends.

In any cervical surgery, anatomical variants must be taken into account. Anatomic path variants are essential to consider, especially the variant in which the IbSLN takes a long loop bilaterally inferior to the greater horn of the hyoid bone before ascending to penetrate the THm, found in 2% of cases [23]. All the more since Kiray stated that IbSLN is most vulnerable near THm [11]. This variation underscores the complexity of the nerve’s course and highlights the importance of meticulous dissection and accurate identification during surgical procedures to prevent nerve injury. Understanding these variations can help improve surgical outcomes and prevent complications associated with damage to the IbSLN.

The IbSLN is the afferent neural pathway for the cough reflex [21]. This reflex is no longer present after the transection of the IbSLN nerve [13]. Additionally, a lesion of the IbSLN results in a loss of sensation in the supraglottis, contributing to difficulty swallowing [14]. Maintaining the integrity of the IbSLN is essential for a proper glottic closure reflex during coughing, vomiting, and swallowing [29,86]. This reflex is necessary for protecting against aspiration of secretions. Damage to the IbSLN can result in loss of sensation in the supraglottic larynx, leading to an increased risk of aspiration and silent aspiration pneumonia [87]. Additionally, damage to the IbSLN can lead to phonatory disorders resulting from dysfunction of the interarytenoid muscle [18,29].

## 13. The IbSLN Nerve Block

The IbSLN provides critical sensory innervation to the supraglottic larynx, base of tongue, epiglottis, and piriform recess [88]. Given its role in mediating the cough and gag reflexes, blocking this nerve has proven highly effective in facilitating a range of upper airway procedures and reducing perioperative complications such as postoperative sore throat and laryngeal discomfort [20,89,90]. In some cases, it can be used for pain relief in the laryngeal area. Complications may occur, such as intravascular injections, nerve damage, dysphagia, blindness, or upper cranial nerve neuropathies [91].

An IbSLN block is frequently performed for awake intubation in patients with difficult airways, such as those with head and neck tumours or cervical spine injury [92]. Bilateral IbSLN blocks can reduce the cardiovascular response caused by the sympathetic stimulation during surgery and reduce the incidence and severity of postoperative cough, sore throat, and hoarseness of voice [93]. An IbSLN block may be used for peroral endoscopy (including laryngoscopy, bronchoscopy, esophagoscopy, and gastroscopy), transesophageal echocardiography, laryngeal and oesophageal instrumentation, laryngography and bronchography, and tracheobronchial toilet [94].

Usually, this block is done blindly by recognising the GhHb and the superior horn of the thyroid cartilage as anatomic landmarks [92]. Several techniques for the transcutaneous IbSLN block have been described and can be categorised into two broad groups: either the injection is intentionally made deep to the THm, or it is made presumably external to the THm [94]. Due to the improvement in ultrasound resolution, it is now possible to use ultrasound guidance for an IBSLN block [92].

Regarding the ultrasound-guided IbSLN nerve block, some authors claim that IbSLN can be accurately identified and localised with ultrasound guidance [22,91,95]. Others encountered difficulties in its identification [96,97]. In such cases, the GhHb is used as a palpable landmark for regional anaesthesia techniques targeting the IbSLN, thereby enhancing the efficacy and safety of the procedure [19,98]. Other landmarks may also be visualised during an ultrasound-guided IbSLN block, such as the THm and the SLA [4,22,99]. The THm appears as a hyperechoic line graph, and the nerve structure around the superior laryngeal artery is the IbSLN [93]. Identifying the external carotid artery and the STA helps identify the SLA [99]. Recent studies have demonstrated the efficacy of ultrasound-guided IbSLN block as a targeted therapeutic approach for chronic neurogenic cough (CnC), a sensory neuropathy [100,101]. It has been suggested that the mechanism of CnC is due to virus-induced damage to the IbSLN [102,103]. The procedure is safe and complication-free [104]. IbSLN steroid injections are also used for the treatment of CnC, with promising results [105,106].

## 14. Conclusions

The IbSLN plays a vital role in the sensory innervation of the supraglottic larynx and significantly contributes to protective reflexes, such as coughing and swallowing. The IbSLN block is of utmost importance in anaesthesiology and in managing the CnC. As anatomical variations may occur, a meticulous surgical dissection is recommended when approaching the THm. The branches of the SLNM should be isolated and spared during approaches nearing the carotid axis.

## Figures and Tables

**Table 1:** The cervical level of the internal branch of the superior laryngeal nerve (IbSLN) origin from the superior laryngeal nerve (SLN).

Cervical Level of the IbSLN Origin from the SLN	Prevalence	Sample	Reference
C1	21%	12 cadavers	[11]
C2	58%	12 cadavers	[11]
C2–C3 intervertebral disk	21%	12 cadavers	[11]
C2	unspecified	25 cadavers	[26]

**Table 2:** Distance to different landmarks of the internal branch of the superior laryngeal nerve entry point through the thyrohyoid membrane. STA: superior thyroid artery.

Landmark	Distance (cm)	Observation	Reference
Greater horn of the hyoid bone	1.65 ± 0.38	No significant gender difference	[33]
	1.59 ± 0.59		[37]
Midpoint of the hyoid body	2.04 ± 0.30	No significant gender difference	[33]
Inferior border of the hyoid bone	0.93 ± 0.16		[30]
Genu of the hyoid bone	average 0.7 (range 0.4–1.1)		[6]
Carotid bifurcation	1.67 ± 0.32	Significantly longer in males (1.94 cm) than in females (1.56 cm), *p* = 0.012	[33]
	2.07 ± 0.59		[37]
Common carotid artery	7.0 ± 6.3		[11]
Superior border of thyroid cartilage	6.0 ± 1.4	No significant gender difference	[33]
	varying from 0 to 1.8	Most frequently (87.5% of cases), the distance varied between 0.1 and 1.2 cm	[18]
	1.2 ± 0.26		[30]
Midpoint of the thyroid notch	1.97 ± 0.29	Significantly higher in males, 2.10 ± 0.19 SD cm in males versus 1.88 ± 0.24 SD cm in female cadavers (*p* = 0.034)	[33]
Laryngeal prominence	13.7 ± 2.8		[11]
	3.29 ± 0.47		[37]
STA origin	1.63 ± 0.42		[37]
	0.59 ± 0.49		[28]
Posterior border of the thyrohyoid muscle	ranging from 0.0 to 1.6	In 80.56% ranged from 0.1 to 0.9	[18]

**Table 3:** The entry of the internal branch of the superior laryngeal nerve (IbSLN) through the thyrohyoid membrane relative to cervical spine levels.

Sample Size	Cervical Level of Penetration Point	Frequency	Reference
20 IbSLN from 10 cadavers	C3–C4 intervertebral disc	Not specified	[6]
24 IbSLN from 12 cadavers	C4	50%	[11]
	C4–C5 intervertebral disc	25%	
	C5	25%	
50 IbSLN from 25 cadavers	C4	Not specified	[26]

**Table 4:** The branching pattern of the internal branch of the superior laryngeal nerve (IbSLN) and the relations to the thyrohyoid membrane (THm).

Study Type	Sample Size	Branching Pattern	Site of Branching	Pre-THm (%)	Post-THm (%)	Reference
Intraoperatively	29 IbSLN of 15 patients	Trifurcation (58.6%), bifurcation (41.4%)	Mixed (pre-THm and post-THm)	4	96	[30]
Cadaveric	22 IbSLN of 12 larynges	Bifurcation (10%), trifurcation (80%), quadrifurcation (15%), pentafurcation (5%)	Post-THm	0	100	[44]
Cadaveric	10 IbSLN of 5 larynges	Trifurcation	Post-THm	0	100	[45]
Cadaveric	24 IbSLNs of 12 larynges	Trifurcation	Mixed (Pre-THm and Post-THm)	37.5	62.5	[11]
Cadaveric	36 cadavers	Trifurcation (72.22%), bifurcation (27.78%)	Pre-THm	100	0	[18]
Cadaveric	25 IbSLN of 19 larynges	Trifurcation	Mixed	84	16	[21]
Cadaveric	25 larynges	Trifurcation	Mixed	14	76	[26]
				in 10%, the nerve could not be traced		

**Table 5:** The length of the internal branch of the superior laryngeal nerve (IbSLN).

IbSLN Length (mm)	Observation	Study Type	Sample	Reference
64	Average length	cadaveric	44 halved heads	[70]
44.9 ± 1.0		cadaveric	50 cadaveric	[23]
57.2 ± 7.7		cadaveric	12 cadavers/24 sides	[11]
6.95 ± 3.71	Length of the IbSLN from the GhHb to the branching point	cadaveric	21 specimens	[21]
23.4 ± 6.9	Chinese	cadaveric	17 cadavers/132 sides	[71]

**Table 6:** The diameter of the internal branch of the superior laryngeal nerve (IbSLN).

IbSLN Diameter (mm)	Observation	Study Type	Sample	Reference
1.8–2.00		cadaveric		[21]
2.1 ± 0.2	at C3 level	cadaveric	24 specimens from 12 cadavers	[11]

## References

[1] Neuhuber W.L., Berthoud H.R. (2022). Functional anatomy of the vagus system: How does the polyvagal theory comply?. Biol. Psychol..

[2] Liu L., Ma Y., Saleh E., Qiu T., Zhuang P. (2024). Exploring the Clinical Characteristics of Superior Laryngeal Nerve Injury. J. Voice.

[3] Yoshida Y., Tanaka Y., Hirano M., Nakashima T. (2000). Sensory innervation of the pharynx and larynx. Am. J. Med..

[4] Liu X., Wang A., Jiao Z., Yao J., Chen X., Luo L., Zhang H. (2024). Effect of ultrasound-guided internal branch of superior laryngeal nerve block on postoperative sore throat induced by a NIM-EMG-ETT: Study protocol for a double-blinded randomized controlled trial. Trials.

[5] Chen H., Xu K., Peng X., Min X. (2024). Key points for protecting the external branch of the superior laryngeal nerve in open thyroidectomy: A possible exploration technique. Surg. Oncol..

[6] Melamed H., Harris M.B., Awasthi D. (2002). Anatomic considerations of superior laryngeal nerve during anterior cervical spine procedures. Spine.

[7] Standring S., Anand N., Birch R., Collins P., Crossman A., Gleeson M., Jawaheer G., Smith A.L., Spratt J.D., Stringer M.D. (2016). Gray’s Anatomy: The Anatomical Basis of Clinical Practice.

[8] Furlan J.C. (2002). Sympathetic fiber origin of the superior laryngeal nerve and its branches: An anatomic study. Clin. Anat..

[9] Kambic V., Zargi M., Radsel Z. (1984). Topographic anatomy of the external branch of the superior laryngeal nerve. Its importance in head and neck surgery. J. Laryngol. Otol..

[10] Jia J., Zhang J., Zeng Z., Shen H., Wang C., Chen J., Xiao S. (2022). Clinical anatomy of superior laryngeal artery via transoral approach. Laryngoscope Investig. Otolaryngol..

[11] Kiray A., Naderi S., Ergur I., Korman E. (2006). Surgical anatomy of the internal branch of the superior laryngeal nerve. Eur. Spine J..

[12] Erfan S., Saha S., Guha R., Sen I., Kulkarni S. (2024). Varying Course of External Branch of Superior Laryngeal Nerve (EBSLN) and Recurrent Laryngeal Nerve (RLN) in Thyroidectomy-An Observational Study. Indian J. Otolaryngol. Head Neck Surg..

[13] Widdicombe J.G., Tatar M. (1988). Upper airway reflex control. Ann. N. Y. Acad. Sci..

[14] Orestes M.I., Chhetri D.K. (2014). Superior laryngeal nerve injury: Effects, clinical findings, prognosis, and management options. Curr. Opin. Otolaryngol. Head Neck Surg..

[15] Sun W., Wen W.P., Zhu X.L. (2022). Preservation of Internal Branch of Superior Laryngeal Nerve during Surgery for Hypopharyngeal Cancer. Ear Nose Throat J..

[16] Martin-Oviedo C., Maranillo E., Sanudo J.R., Perez-Lloret P., Verdu E., Martinez-Guirado T., Alvarez-Montero O., Gomez Martin-Zarco J.M., Vazquez T. (2019). The Human Laryngeal Innervation Revisited—The Role of the Neural Connections. Anat. Rec..

[17] Ahmad R., Saraf A., Kishore K., Kalsotra P. (2022). Relation of Superior Laryngeal Nerve and Superior Thyroid Artery with Superior Pole of Thyroid During Thyroid Surgery. Indian J. Otolaryngol. Head Neck Surg..

[18] Paraskevas G.K., Raikos A., Ioannidis O., Brand-Saberi B. (2012). Topographic anatomy of the internal laryngeal nerve: Surgical considerations. Head Neck.

[19] El Deek A.M., Shafik A.M., Eltohry A.S.M.A., Al Fawal S.M. (2021). Comparison between ultrasound-guided and anatomical landmark-guided block of internal branch of the superior laryngeal nerve for awake fiber-optic intubation in suspected difficult intubation: A randomized controlled study. Ain-Shams J. Anesthesiol..

[20] Zhipeng L., Meiyi H., Meirong W., Qunmeng J., Zhenhua J., Yuezhen H., Jinfang Z., Chuiliang L. (2020). Ultrasound-guided internal branch of superior laryngeal nerve block on postoperative sore throat: A randomized controlled trial. PLoS ONE.

[21] Stephens R.E., Wendel K.H., Addington W.R. (1999). Anatomy of the internal branch of the superior laryngeal nerve. Clin. Anat..

[22] Stopar-Pintaric T., Vlassakov K., Azman J., Cvetko E. (2015). The thyrohyoid membrane as a target for ultrasonography-guided block of the internal branch of the superior laryngeal nerve. J. Clin. Anesth..

[23] Furlan J.C., Brandao L.G., Ferraz A.R., Rodrigues A.J. (2003). Surgical anatomy of the extralaryngeal aspect of the superior laryngeal nerve. Arch. Otolaryngol. Head Neck Surg..

[24] Shin D.U., Sung J.K., Nam K.H., Cho D.C. (2012). Bilateral internal superior laryngeal nerve palsy of traumatic cervical injury patient who presented as loss of cough reflex after anterior cervical discectomy with fusion. J. Korean Neurosurg. Soc..

[25] Hill J.H., Olson N.R. (1979). The surgical anatomy of the spinal accessory nerve and the internal branch of the superior laryngeal nerve. Laryngoscope.

[26] Bharathadevi M.S.T. (2019). A cadaveric study of internal branch of superior laryngeal nerve. Int. J. Sci. Res..

[27] Monfared A., Kim D., Jaikumar S., Gorti G., Kam A. (2001). Microsurgical anatomy of the superior and recurrent laryngeal nerves. Neurosurgery.

[28] Ozgur Z., Govsa F., Celik S., Ozgur T. (2009). Clinically relevant variations of the superior thyroid artery: An anatomic guide for surgical neck dissection. Surg. Radiol. Anat..

[29] Dekhou A.S., Morrison R.J., Gemechu J.M. (2021). The Superior Laryngeal Nerve and Its Vulnerability in Surgeries of the Neck. Diagnostics.

[30] Guven E.M., Karacan K., Guven M., Elden H., Ozcelik Korkmaz M. (2021). Topographic anatomy of the internal branch of the superior laryngeal nerve. Eur. Arch. Oto-Rhino-Laryngol..

[31] Calota R.N., Rusu M.C., Rusu M.I., Dumitru C.C., Vrapciu A.D. (2025). Anatomical Variables of the Superior Thyroid Artery on Computed Tomography Angiograms. Medicina.

[32] Chandrasekhar S.S., Randolph G.W., Seidman M.D., Rosenfeld R.M., Angelos P., Barkmeier-Kraemer J., Benninger M.S., Blumin J.H., Dennis G., Hanks J.J.O.H. (2013). Clinical practice guideline: Improving voice outcomes after thyroid surgery. Otolaryngol. Head Neck Surg..

[33] Turk B., Canda B., Pence K.B., Yuzbasioglu N., Turgut S. (2023). Surgical landmarks for identification and preservation of the internal branch of the superior laryngeal nerve. Surg. Radiol. Anat..

[34] Uludağ M., Tanal M., İşgör A. (2018). A review of methods for the preservation of laryngeal nerves during thyroidectomy. J. Clin. Anesth..

[35] Sulica L. (2004). The superior laryngeal nerve: Function and dysfunction. Otolaryngol. Clin. N. Am..

[36] Dilworth T.F. (1921). The Nerves of the Human Larynx. J. Anat..

[37] Devaraja K., Punja R., Kalthur S.G., Pujary K. (2021). Unmapped landmarks around branches of the Superior Laryngeal Nerve: An exploratory cadaveric study. J. Taibah Univ. Med. Sci..

[38] Poutoglidis A., Paraskevas G.K., Lazaridis N., Anastasopoulos N., Asouhidou I., Argyroulis A., Galanis N., Karamitsou P., Paraskevas G.J.C. (2023). Bilateral Thyroid Foramina in a Completely Ossified Laryngeal Framework: A Case Report. Cureus.

[39] Srichaphan N., Yurasakpong L., Taradolpisut N., Senarai T., Kruepunga N., Suwannakhan A. (2024). The thyroid foramen: A systematic review and meta-analysis. Surg. Radiol. Anat..

[40] Leon X., Maranillo E., Mirapeix R.M., Quer M., Sanudo J.R. (1997). Foramen thyroideum: A comparative study in embryos, fetuses, and adults. Laryngoscope.

[41] Ramsaroop L., Hurrinarain K., Partab P., Satyapal K.S. (2010). The incidence of the foramen thyroideum in the South African population. Int. J. Morphol..

[42] He X., Ye C., Carrat X., Traissac L. (1999). Anatomical study of the thyroid foramen in human larynx: A study of 100 dissections. Rev. Laryngol. Otol. Rhinol. (Bord).

[43] Sanudo J.R., Maranillo E., Leon X., Mirapeix R.M., Orus C., Quer M. (1999). An anatomical study of anastomoses between the laryngeal nerves. Laryngoscope.

[44] Rueger R.S. (1972). The superior laryngeal nerve and the interarytenoid muscle in humans: An anatomical study. Laryngoscope.

[45] Sanders I., Mu L. (1998). Anatomy of the human internal superior laryngeal nerve. Anat. Rec. A Discov. Mol. Cell Evol. Biol..

[46] Paraskevas G.K., Poutoglidis A., Lazaridis N., Anastasopoulos N., Tsetsos N. (2023). Early Internal Branch of Superior Laryngeal Nerve Bifurcation Passes Through Double Thyroid Foramen. Ear Nose Throat J..

[47] Kim D.W., Lee H., Ji J.Y., Mohammad R.T., Huh G., Jeong W.J., Cha W. (2023). Superior Laryngeal Nerve Block in Transcutaneous Vocal Fold Injection: A Pilot Study. J. Voice.

[48] Lemere F.J.T.A.R. (1932). Innervation of the larynx. II. Ramus anastomoticus and ganglion cells of the superior laryngeal nerve. Anat. Rec..

[49] Santoso L.F., Jafari S., Kim D.Y., Paydarfar D. (2021). The Internal Superior Laryngeal Nerve in Humans: Evidence for Pure Sensory Function. Laryngoscope.

[50] Pascual-Font A., Cubillos L., Vazquez T., McHanwell S., Sanudo J.R., Maranillo E. (2016). Are the interarytenoid muscles supplied by branches of both the recurrent and superior laryngeal nerves?. Laryngoscope.

[51] Mu L., Sanders I., Wu B.L., Biller H.F. (1994). The intramuscular innervation of the human interarytenoid muscle. Laryngoscope.

[52] Kotby M.N., Haugen L.K. (1970). Attempts at evaluation of the function of various laryngeal muscles in the light of muscle and nerve stimulation experiments in man. Acta Oto-Laryngol..

[53] Ziegelman E.F. (1933). Laryngeal nerves: Surgical importance in relation to the thyroid arteries, thyroid gland and larynx. Arch. Otolaryngol. Neck Surg..

[54] Sakamoto Y. (2013). Interrelationships between the innervations from the laryngeal nerves and the pharyngeal plexus to the inferior pharyngeal constrictor. Surg. Radiol. Anat..

[55] Berger G., Kosztyla-Hojna B., Chyczewski L. (2018). Impact of the anatomy of laryngeal nerves on intraoperative neuromonitoring results in surgery of thyroid gland and functional results after partial laryngectomies. Pol. Przegl. Chir..

[56] Kreyer R., Pomaroli A. (2000). Anastomosis between the external branch of the superior laryngeal nerve and the recurrent laryngeal nerve. Clin. Anat..

[57] Hisa Y. (2016). Neuroanatomy and Neurophysiology of the Larynx.

[58] Kochilas X., Bibas A., Xenellis J., Anagnostopoulou S. (2008). Surgical anatomy of the external branch of the superior laryngeal nerve and its clinical significance in head and neck surgery. Clin. Anat..

[59] Hammer G.P., Tomazic P.V., Vasicek S., Graupp M., Gugatschka M., Baumann A., Konstantiniuk P., Koter S.H. (2016). Carotid endarterectomy significantly improves postoperative laryngeal sensitivity. J. Vasc. Surg..

[60] Uluisik I.E., Elbizim D.S., Ortug G. (2023). A Microdissectional Study on Galen’s Anastomosis: Anatomical Perspective with Clinical Emphasis. J. Anat. Soc. India.

[61] Ónodi A. (1902). Die Anatomie und Physiologie der Kehlkopfnerven: Mit Ergänzenden Pathologischen Beiträgen.

[62] Stefanou C.K., Papathanakos G., Stefanou S.K., Tepelenis K., Kitsouli A., Barbouti A., Tsoumanis P., Kanavaros P., Kitsoulis P. (2022). Surgical tips and techniques to avoid complications of thyroid surgery. Innov. Surg. Sci..

[63] Henry B.M., Pekala P.A., Sanna B., Vikse J., Sanna S., Saganiak K., Tomaszewska I.M., Tubbs R.S., Tomaszewski K.A. (2017). The Anastomoses of the Recurrent Laryngeal Nerve in the Larynx: A Meta-Analysis and Systematic Review. J. Voice.

[64] Maranillo E., Leon X., Orus C., Quer M., Sanudo J.R. (2005). Variability in nerve patterns of the adductor muscle group supplied by the recurrent laryngeal nerve. Laryngoscope.

[65] Partab P., Hurrinarain K., Ramsaroop L., Satyapal K.S. (2006). Atypical anastomosis of laryngeal nerves. Clin. Anat..

[66] Dubey R., Raj P. (2020). Superior laryngeal nerve dysfunction following thyroid surgery: Assessment of incidence and long-term effect. Int. J. Otorhinolaryngol. Head Neck Surg..

[67] Lombardi C.P., Raffaelli M., D’Alatri L., Marchese M.R., Rigante M., Paludetti G., Bellantone R. (2006). Voice and swallowing changes after thyroidectomy in patients without inferior laryngeal nerve injuries. Surgery.

[68] Ma L., Ren H.C., Huang Y., Yin L. (2024). Hybrid Surgery for Revascularization of Chronic Occlusion of Internal Carotid Artery. J. Craniofac. Surg..

[69] Tempel Z.J., Smith J.S., Shaffrey C., Arnold P.M., Fehlings M.G., Mroz T.E., Riew K.D., Kanter A.S. (2017). A Multicenter Review of Superior Laryngeal Nerve Injury Following Anterior Cervical Spine Surgery. Glob. Spine J..

[70] Lang J., Nachbaur S., Fischer K., Vogel E. (1987). The superior laryngeal nerve and the superior laryngeal artery. Acta Anat..

[71] Lu K.N., Ding J.W., Zhang Y., Shi J.J., Zhou L., Peng Y., Shen J., Lu S., Sun S.H., Ni Y.Q. (2021). The Anatomical and Clinical Significance of the Superior Laryngeal Nerve. Otolaryngol. Head Neck Surg..

[72] Wasserman J.M., Sundaram K., Alfonso A.E., Rosenfeld R.M., Har-El G. (2008). Determination of the function of the internal branch of the superior laryngeal nerve after thyroidectomy. Head Neck.

[73] Potenza A.S., Araujo Filho V.J.F., Cernea C.R. (2017). Injury of the external branch of the superior laryngeal nerve in thyroid surgery. Gland. Surg..

[74] Bourabaa S., Settaf A. (2024). Is identification and dissection of the external laryngeal nerve necessary during thyroidectomy? A prospective study. BMC Surg..

[75] Singh K., Singh I., Meher R., Kumar J., Gopal A., Sahoo A., Sharma R. (2025). Incidence of Injury to External Branch of Superior Laryngeal Nerve as Diagnosed by Acoustic Voice Analysis After Thyroidectomy. Indian J. Otolaryngol. Head Neck Surg..

[76] Barman S., Bhattacharjee A., Mahanta B., Nath K. (2025). Role of Intraoperative Neuromonitoring to Identify External Branch of Superior Laryngeal Nerve During Thyroidectomy. Indian J. Otolaryngol. Head Neck Surg..

[77] Yu X., Zhu R., Zhu P., Du Y., Tanu C., Han Z., Jiang N., Pan L., Xie C., Zhao Q. (2025). Effectiveness and feasibility of nerve real-time monitoring and intermittent monitoring in endoscopic thyroidectomy: A multicenter retrospective cohort study of 1621 patients. Int. J. Surg..

[78] Cernea C.R., Brandao L.G., Hojaij F.C., De Carlucci D., Montenegro F.L., Plopper C., Vanderlei F., Gotoda R., Dias F.L., Lima R.A. (2010). How to minimize complications in thyroid surgery?. Auris Nasus Larynx.

[79] Cernea C.R., Ferraz A.R., Nishio S., Dutra A., Hojaij F.C., dos Santos L.R. (1992). Surgical anatomy of the external branch of the superior laryngeal nerve. Head Neck.

[80] Cernea C.R., Nishio S., Hojaij F.C. (1995). Identification of the external branch of the superior laryngeal nerve (EBSLN) in large goiters. Am. J. Otolaryngol..

[81] Friedman M., LoSavio P., Ibrahim H. (2002). Superior laryngeal nerve identification and preservation in thyroidectomy. Arch. Otolaryngol. Head Neck Surg..

[82] Dip F., Aleman R., Marinelli F., Guiselli J., Rosenthal R., Rancati A., Sinagra D. (2025). High-Precision Identification of Sensory and Motor Branches of the Recurrent Laryngeal Nerve Via Autofluorescence System in Thyroid Surgery. Cureus.

[83] Yadav S.K., Agarwal P., Sharma D. (2025). Critical View of Safety: Anatomical Key to Avoid Injury to Recurrent Laryngeal Nerve in Transoral Endoscopic Thyroidectomy. Laryngoscope.

[84] Yao X.Y., Li X., Yu B., Liu S.R., Wang B.Y., Lu S.Y., Li H.W., Song S.B., Cui L.G., Tan S. (2025). Ultrasound Visualization of the Recurrent Laryngeal Nerve: A Prospective Clinical Validation Study. Ann. Surg. Oncol..

[85] Kafki S., Chrysikos D., Manoli A., Troupis T. (2023). Anatomical Variations of the External Branch of the Superior Laryngeal Nerve and its Correlations with the Superior Thyroid Artery and the Upper Pole of the Thyroid Gland: A Review of the Literature. J. Surg. Surg. Spec..

[86] Edmonds C.E., German R.Z., Bond L.E., Mayerl C.J. (2022). Oropharyngeal capsaicin exposure improves infant feeding performance in an animal model of superior laryngeal nerve damage. J. Neurophysiol..

[87] Rice D.H. (1982). Laryngeal reinnervation. Laryngoscope.

[88] Kang Q., Wu L., Liu Y., Zhang X. (2023). Ultrasound-guided medial branch of the superior laryngeal nerve block to reduce peri-operative opioids dosage and accelerate patient recovery. PLoS ONE.

[89] Chen Z., Jin Y., Lu G., Jin Y., Feng C., Zhao X. (2023). Preoperative Ultrasound-Guided Internal Branch Block of Superior Laryngeal Nerve Reduces Postoperative Sore Throat Caused by Double Lumen Endotracheal Intubation: A Randomized Trial. Anesth. Analg..

[90] Jin Y., Zhou X., Chen X., Cai J., Zhao Q., Huang X., Pan Y., Sun J. (2022). Internal branch of superior laryngeal nerve block by dexamethasone alleviates sore throat after thyroidectomy: A randomized controlled trial. Eur. Arch. Oto-Rhino-Laryngol..

[91] Bao Y., Xiong J., Wang H., Zhang Y., Zhong Q., Wang G. (2022). Ultrasound-Guided Block of the Internal Branch of the Superior Laryngeal Nerve Reduces Postoperative Sore Throat Caused by Suspension Laryngoscopic Surgery: A Prospective Randomized Trial. Front. Surg..

[92] Lan C.-H., Cheng W.-C., Yang Y.-L. (2013). A new method for ultrasound-guided superior laryngeal nerve block. Tzu Chi Med. J..

[93] Zhou Y., Chen B., Xiong Y., Yu X. (2022). The Efficacy of Ultrasound-Guided Superior Laryngeal Nerve Block as an Adjuvant to General Anesthesia during Suspension Laryngoscopy Vocal Cord Polypectomy. Evid. Based Complement. Altern. Med..

[94] Stockwell M., Lozanoff S., Lang S.A., Nyssen J. (1995). Superior laryngeal nerve block: An anatomical study. Clin. Anat..

[95] Kaur B., Tang R., Sawka A., Krebs C., Vaghadia H. (2012). A method for ultrasonographic visualization and injection of the superior laryngeal nerve: Volunteer study and cadaver simulation. Anesth. Analg..

[96] Vaghadia H., Lawson R., Tang R., Sawka A. (2011). Failure to visualise the superior laryngeal nerve using ultrasound imaging. Anaesth. Intensive Care.

[97] Green J.S., Tsui B.C. (2010). Applications of ultrasonography in ENT: Airway assessment and nerve blockade. Anesth. Clin..

[98] Talbot N., Heller M., Nyirjesy S., Kim B., DeSilva B., Matrka L. (2023). Superior Laryngeal Nerve Block Response Rates in 54 Neurogenic Cough Patients. Laryngoscope.

[99] Manikandan S., Neema P.K., Rathod R.C. (2010). Ultrasound-guided bilateral superior laryngeal nerve block to aid awake endotracheal intubation in a patient with cervical spine disease for emergency surgery. Anesthesiol. Intensive Care.

[100] Simpson C.B., Tibbetts K.M., Loochtan M.J., Dominguez L.M. (2018). Treatment of chronic neurogenic cough with in-office superior laryngeal nerve block. Laryngoscope.

[101] Quinton B.A., Tierney W.S., Benninger M.S., Nelson R.C., Gau V.L., Hrelec C.M., Bryson P.C. (2024). The Role of Bilateral Superior Laryngeal Nerve Block in Managing Refractory Chronic Cough. Laryngoscope.

[102] Campagnolo A., Nickel V., Benninger M.S. (2025). Efficacy and Safety of Superior Laryngeal Nerve Block in the Management of Neuropathic Cough: A Systematic Review. Lung.

[103] Dhillon V.K. (2019). Superior laryngeal nerve block for neurogenic cough: A case series. Laryngoscope Investig. Otolaryngol..

[104] Chen Z., Zhang L., Lu G., Zhang Y., Zhao D., Zhao S., Zhang H., Jin Y., Zhao X., Jin Y. (2025). Effects of Dexmedetomidine as an Adjuvant in Preoperative Ultrasound-Guided Internal Branch of Superior Laryngeal Nerve Block on Postoperative Sore Throat and Hemodynamics in Patients With Double-Lumen Endotracheal Intubation: A Randomized Controlled Trial. J. Pain Res..

[105] Bowen A.J., Roitman A., Ring S., Francis D.O., Davis R.J., McCulloch T., Dailey S.H. (2025). Bilateral internal superior laryngeal nerve injections for unexplained chronic cough. Am. J. Otolaryngol..

[106] Asi K., Vance D.G., Tritter A.G. (2025). Survey of Practice Patterns in the Use of Superior Laryngeal Nerve Blocks. Laryngoscope.

